# Hydrogen Storage with Aluminum Formate, ALF: Experimental, Computational, and Technoeconomic Studies

**DOI:** 10.1063/4.0000879

**Published:** 2025-10-27

**Authors:** Hayden Evans, Taner Yildirim, Peng Peng, Yongqiang Cheng, Zeyu Deng, Qiang Zhang, Dinesh Mullangi, Dan Zhao, Pieremanuele Canepa, Hanna Breunig, Anthony K Cheetham, Craig M Brown

**Affiliations:** 1 NIST, Gaithersburg, MD, USA; 2 Lawrence Berkeley National Laboratory, Berkeley, CA, USA; 3 ORNL, Oak Ridge, TN, USA; 4 NUS, Singapore, Singapore, Singapore; 5 UH, Houston, TX, USA; 6 UCSB, Santa Barbara, CA, USA

## Abstract

Long-duration storage of hydrogen is necessary for coupling renewable H_2_ with stationary fuel cell power applications. In this presentation, I will discuss how aluminum formate, Al(HCOO)_3_ (ALF), which adopts an ReO_3_-type structure, is shown to have remarkable H2 storage performance at non-cryogenic (> 120 K) temperatures and low pressures. The most promising performance of ALF is found between 120 K and 160 K and at 10 bar to 20 bar. The talk will cover and illustrate the H2 adsorption performance of ALF over the 77 K to 296 K temperature range using gas isotherms, in situ neutron powder diffraction, and DFT calculations, as well as technoeconomic analysis (TEA), illustrating ALF’s competitive performance for long-duration storage versus compressed hydrogen and leading metal–organic frameworks. In the TEA, it is shown that ALF’s storage capacity, when combined with a temperature/pressure swing process, has advantages versus compressed H_2_ at a fraction of the pressure (15 bar versus 350 bar). Given ALF’s performance in the 10 bar to 20 bar regime under moderate cooling, it is particularly promising for use in safe storage systems serving fuel cells, and is currently the only MOF that works in this moderate temperature range/ low pressure regime to be cost competitive with compressed H_2_ gas for large scale H_2_ storage.^[1]^
